# Physical Functioning in Patients with a Recent Fracture: The “Can Do, Do Do” Framework Applied to Explore Physical Capacity, Physical Activity and Fall Risk Factors

**DOI:** 10.1007/s00223-023-01090-3

**Published:** 2023-06-27

**Authors:** M. R. Schene, K. Meijer, D. Cheung, H. C. Willems, J. H. M. Driessen, L. Vranken, J. P. van den Bergh, C. E. Wyers

**Affiliations:** 1 NUTRIM School of Nutrition and Translational Research in Metabolism, Maastricht University, Maastricht, The Netherlands; 2 Department of Internal Medicine, VieCuri Medical Center, P.O. Box 1926, 5900 BX Venlo, The Netherlands; 3 Internal Medicine and Geriatrics, Amsterdam UMC Location University of Amsterdam, Meibergdreef 9, Amsterdam, The Netherlands; 4 Department of Nutrition and Movement Sciences, Maastricht University, Maastricht, The Netherlands; 5 Amsterdam Bone Center, Movement Sciences Amsterdam, Amsterdam, The Netherlands; 6 Department of Clinical Pharmacy and Toxicology, Maastricht University Medical Centre+, Maastricht, The Netherlands; 7 CARIM School of Cardiovascular Research Institute Maastricht, Maastricht University, Maastricht, The Netherlands; 8 Department of Internal Medicine, Maastricht University Medical Center +, P.O. Box 616, 6200 MD Maastricht, The Netherlands

**Keywords:** Fracture liaison service1, Physical activity2, Physical capacity3, Falls4, Accelerometer5

## Abstract

Physical capacity (PC) and physical activity (PA) are associated physical performance measures, and combined, PC and PA are used to categorize physical performance in the “can do, do do” framework. We aimed to explore physical performance of patients attending the fracture liaison service (FLS). In this cross-sectional study, PC was measured by 6-min-walking-test (can’t do/can do) and PA by accelerometer (don’t do/do do). Following quadrants were defined based on predefined cut-off scores for poor performance: (1) “can’t do, don’t do”; (2) “can do, don’t do”; (3) “can’t do, do do”; (4) “can do, do do”. Odds ratios (OR) were calculated and fall and fracture risk factors were assessed between quadrants. Physical performance of 400 fracture patients was assessed (mean age 64; female 70.8%). Patients performed as follows: 8.3% “can’t do, don’t do”; 3.0% “can do, don’t do”; 19.3% “can’t do, do do”; 69.5% “can do, do do”. For the “can’t do” group the OR for low PA was 9.76 (95% CI: 4.82–19.80). Both the “can’t do, don’t do” and “can’t do, do do” group differed significantly compared to the “can do, do do” group on several fall and fracture risk factors and had lower physical performance. The “can do, do do” framework is able to identify fracture patients with an impaired physical performance. Of all FLS patients 20% “can’t do, but “do do” while having a high prevalence of fall risk factors compared to persons that “can do, do do”, which may indicate this group is prone to fall.

**Supplementary Information:**

The online version contains supplementary material available at 10.1007/s00223-023-01090-3.

## Introduction

Approximately 30% of people aged 65 and older fall at least once a year [1]. The health burden of falls is considerable, as 40–60% of falls result in injury [2]. About 5% percent of all falls result in a fracture. Conversely, 70% to 90% of all fractures are caused by a fall [3]. Patients with a previous fracture have an increased risk of sustaining a subsequent fracture [4]. Fracture Liaison Services (FLSs) have been implemented to identify, evaluate and treat patients with an increased risk of subsequent fractures [5]. FLS evaluation includes assessment of comorbidities and medication use, screening for osteoporosis (including bone mineral density and imaging of the spine) and underlying contributors for secondary osteoporosis and other metabolic bone disease [6]. Although, in a recent meta-analyses it was shown that the FLS approach results in a significant lower probability of 30% for subsequent fracture risk within two years follow-up [7], still, approximately 8% of fracture patients sustain a subsequent fracture, indicating need for additional preventative measures [7]. Ideally, FLS screening also includes a fall risk assessment [5].The prevalence of fall risk factors in FLS patients is high [8, 9] and as fall risk is potentially modifiable, optimizing strategies for early fall prevention in FLS patients is essential [5].

An important part of fall risk evaluation is the assessment of physical functioning. Physical capacity (PC) and physical activity (PA) are two associated, but distinctly separate domains of physical functioning [10]. PC is objectively measured physical functioning with specific tests such as the six-minute walking test (6MWT) [11], while PA is ‘any bodily movement produced by skeletal muscles that results in energy expenditure above resting level’ and is measured during daily life [12]. PA is recommended by the WHO because of its benefits on a wide range of health outcomes [13]. Both PC and PA measures have been associated with falls. Poor performance on physical capacity tests is associated with an increased fall risk [14, 15]. Importantly, low PC is potentially modifiable; exercise intervention to improve strength and balance reduce the rate of falls among older persons by approximately 25% [16]. The association between physical activity and falls is not yet fully comprehended. Some studies report an association between an increased PA and fewer falls [16–18]. Others report that higher physical activity is related to a higher rate of falls, possibly due to a higher exposure of risks [16, 19], as about 40–60% of falls in older persons occur during walking [20]. Lu et al. hypothesize that the association between PA and falls is U-shaped, implying that both inactive and highly active older adults have higher fall rates [21]. Combined evaluation of performance on PC and PA measures might provide additional insight on physical functioning and fall risk in fracture patients.

In a recent study, Koolen et al. developed a PC (can’t do or can do) and PA (don’t do or do do) quadrant framework to understand impaired physical function in COPD patients [22]. This framework can be applied to FLS patients; patients who have fallen in the past and are prone to sustain falls in the future [3]. This concept allows for an integrated assessment of physical functioning as well as for the identification of quadrant subgroups with specific clinical characteristics such as modifiable fall risk factors in fracture patients. The aim of this study is to categorize physical performance of patients with a recent clinical fracture attending the FLS using the “can do, do do” framework and to assess the distribution of patients over the PC-PA quadrants and correlation between PC and PA measures. Further, we aim to explore the clinical characteristics and outcome on functional performance tests between quadrant groups.

## Materials and Methods

### Study Design and Participants

This study is designed as a cross-sectional study including baseline data from the FX MoVie study, a prospective cohort study (*n* = 500) conducted at FLS of VieCuri Medical Centre in Venlo, the Netherlands. Included were all patients aged 50–90 years with a recent radiographically confirmed fracture who attended the FLS for fracture risk evaluation and who were able and willing to participate. Excluded were non-Caucasian patients, patients with cognitive impairments, patients who were currently being treated for malignancy, patients with fractures due to a bone metastasis or osteomyelitis, or peri-prosthetic fractures, or fractures due to a high energetic trauma. At the FLS, all patients were evaluated and treated according to Dutch guidelines on osteoporosis and fracture prevention [23]. Patients received information on the study through oral and written communication and gave written informed consent prior to participation. The study protocols were approved by an independent medical ethical committee (NL45707.072.13). Patients with missing 6MWT scores or physical activity data were excluded from the analyses.

### Data Collection

Baseline data include age, sex, height, weight, and body mass index (BMI) measured by trained nurse during clinic visit. Further, at the time of fracture risk evaluation, patients were asked to fill out a detailed questionnaire for evaluation of cause of the fracture, risk factors for falls, fractures, and osteoporosis, medication use, and included self-reported variables such as living situation, smoking and alcohol use, dizziness and balance problems, urinary incontinence, vision impairment, use of walking aids, fear of falling, dizziness or balance problems and number of falls in the past 12 months. Comorbidities and fractures were derived from the electronic patient files and categorized according to ICD-10 standards [24]. Fracture location was grouped into hip, major and minor fractures according to Center et al. [25]: (I) hip fractures, (II) major fractures; vertebra, multiple rib, humerus, pelvis, distal femur and proximal tibia, and (III) minor fractures; all other fractures (including finger and toe fractures). Functional assessments were performed during (PC measures), or just after (PA) the baseline visit and was overseen by trained nurses.

### Measurements of the Quadrant Concept

#### Physical Capacity

The main outcome measure of physical capacity was the six-minute walking distance (6MWD) measured by the 6MWT. 6MWT is a valid test with excellent test–retest and inter-rater reliability [11, 26] and was assessed in a linoleum hallway using standardized instructions [27]. Patients were asked to walk around two safety cones placed 10 m from each other at a comfortable speed while covering as much distance as possible. No encouragements were given during the test. Use of walking aids was permitted. Patients were allowed to rest or stop when needed. 6MWD was calculated by multiplying the number of rounds with 20 m and additionally adding the meters of final lap.

#### Physical Activity

The physical activity of the participants was measured during eight consecutive days of free living using the triaxial accelerometer MOX (Maastricht Instruments B.V., The Netherlands). Details of the processing of raw accelerometer data and activity classification is explained in more detail elsewhere [28, 29]. In short, MOX is a small waterproof device with the dimensions 4.5 × 4.0 × 1.4 cm, a sampling frequency of 25 Hz, sensor range of ± 6 G [29]. The MOXBW software (Maastricht Instruments B.V., The Netherlands) was used to calibrate the device. It was attached to every subject on the right thigh ten centimeters above the knee, by the same researcher. The participants were instructed to follow their normal daily routines, while keeping an activity log. Data of the MOX and activity log of at least six consecutive days, including at least one weekend day, was used for analysis. The raw acceleration data was processed using MATLAB R2012a software (The MathWorks Inc., Natick, MA, USA). Using the signal magnitude area, a measure of the intensity of physical activity, static and dynamic activity (DA) were determined. DA is expressed as average minutes per day and was further categorized as low physical activity (LPA), moderate-to-vigorous physical activity (MVPA), and vigorous physical activity (VPA), respectively corresponding to intensities below 3 metabolic equivalents (METS), between 3 and 6 METS, and above 6 METS, respectively [29].

#### Quadrant Concept and Threshold for Poor Performance

Patients were divided in mutually exclusive categories using the quadrant concept for PC and PA presented by Koolen et al. [22]. (1) Low PC, low PA (can’t do, don’t do); (2) preserved PC, low PA (can do, don’t do); (3) low PC, preserved PA (can’t do, do do); and (4) preserved PC, preserved PA (can do, do do). The threshold for low PC was defined as − 2SD below sex specific means of the 6MWD, using reference values of Beekman et al. [30]; 625 ± 120 m for men and 554 ± 94 m for women. Thus, the threshold for low PC was 385 m for men and 366 m for women, respectively. The threshold of low PA was defined as < 150 min of MVPA/VPA per week (21.4 min/day), as this is the recommended minimum intensity adults and older adults (65 +) should exercise for general health benefits by the WHO [13].

### Other Measures

In addition to the main PC measure, three other functional performance tests were performed; timed up and go (TUG), handgrip strength (HGS) and chair stand test (CST). All tests have proven validity and good to excellent test-retest and inter-rater reliability in elderly populations with and without comorbidities [31–35]. TUG measures balance and overall mobility [31]. Participants were observed and timed while they rise from a chair with armrests, walk for three meters, turn, walk back to the chair and sit down again. Use of walking aids was permitted. Of a total of three attempts, the mean time in seconds was used as TUG score. HGS measures upper extremity strength and was measured by handheld dynamometer (Jamar, Sammons Preston, Bolingbrook, Illinois) [35]. The participant was seated with the elbows flexed at 90 degrees and was asked to squeeze as hard as possible with their left and right hand. The maximum HGS in kg was the highest score out of three attempts for their left hand and right hand and was used for analysis. Lower body muscle strength was assessed by the timed CST [32]. This test measured the number of times a person can fully stand up from a chair in 30 s time. Patients were instructed to start in seated position, cross arms over the chest, fully sit back down in between stands, and not to use the armrests, unless unavoidable. Appendicular Lean Mass (ALM) was measured as the sum of lean mass of arms and legs and corrected for squared height. Sarcopenia was defined following European Working Group on Sarcopenia in Older People (EWGSOP2) guidelines as having low ALM (< 5.5 kg/m2 for women or < 7.0 kg/m2 for men), combined with having either low scores on the 30CST (< 10 stands), and/or low HGS (< 16 kg for women and < 27 kg for men) [36]. Measurement of bone mineral density (BMD) and vertebral fracture assessment (VFA) were carried out. The BMD of the participants was measured with a total body dual energy X-ray absorptiometry (DXA) using the Hologic Discovery DXA system (Hologic Inc, Bedford, MA, USA). A trained radiology technologist scanned the lumbar spine vertebra L1 to L4, total hip, and femoral neck of the left leg. The lowest T-score score of the three measurements was used and normal BMD was defined as a T-score ≥ -1SD, osteopenia was defined as T -score < − 1.0 SD and osteoporosis < − 2.5 SD, in accordance with WHO criteria [37]. Diagnosis of osteosarcopenia was given in case of osteopenia or osteoporosis diagnosis combined with a diagnosis of sarcopenia (EWGSOP2) [38]. Prevalent vertebral fractures (VFs) were assessed on lateral spine images acquired with DXA. Grading of VFs was done morphometrically using the VF classification of Genant [39]. VFs were graded based on % height loss as follows; grade 1 (20 to 25%), grade 2 (25 to 40%), or grade 3 (height loss > 40%). Patients were classified according to the most severe VF.

### Statistical Analysis

Baseline characteristics were described using frequencies (proportions) for categorical variables and means (SD) or medians (interquartile ranges) for continuous data. To examine the relationship between PC and PA measures a Pearson’s correlation coefficient (PCC) was calculated. Strength of correlation was defined as follows; PCC 0.0 to 0.3 negligible; 0.3 to 0.5 low; 0.5 to 0.7 moderate; 0.7 to 0.9 high and 0.9 to 1 very high correlation [40]. Furthermore, we calculated an OR between the PC and PA categories. Analyses were stratified by sex due to the sex specific cut-off scores of the 6MWT. Assessment of differences between quadrants was carried out for all measured variables. Comparison between groups for categorical variables was performed using Chi-Square test or Fisher exact tests (in case of small sample sizes) with post-hoc testing using pairwise Z-test with Bonferroni correction. One-way ANOVA was used for normally distributed continuous variables. Equality of variances was checked and overall tests and post-hoc tests (Bonferroni, or Games-Howell) were performed accordingly. In case of non-normal data, a Kruskall-Wallis test was performed. *p*-values < 0.05 were considered statistically significant. All statistical analyses were conducted using SPSS version 20 (IBM Corp., USA).

## Results

### Study Population

A total of 500 participants out of 1011 consecutive FLS attendees consented to participate in the FX MoVie cohort. One hundred participants were excluded due to missing data on PA or PC measures, resulting in 400 participants available for analysis. Participants attended the FLS on average 3.5 (1.0) months after fracture. Mean age was 64.5 (8.1) years for women and 64.8 (9.7) years for men, and 70.8% of the patients were female. Fracture types for men and women respectively were as follows; 74% and 75% minor fracture, 21% and 20% major fractures and 5% hip fractures in both groups. In men, 83.8% of fractures were caused by a fall, in women 86.5%, respectively. At least 2 falls in the past 12 months, excluding the fall that caused the fracture, were reported by 12% of women and 14.5% of men. Osteoporosis was diagnosed in 24.7% of women and 15.2% of men. In both men and women, at least one prevalent VF grade 2 or 3 was present in 12.0% of participants.

### Functional Performance

Overall, 72.3% of the participants performed in the “can do” group (PC [6MWD] > 385 m for men and > 366 m for women), and 88.8% of the participants were categorized in the “do do” group (PA > 21.4 min/day). This was 74.4% (PC) and 86.9% (PA) in women and 74.4% (PC) and 94.9% (PA), in men, respectively. Of all participants in the “can do” group, 95.8% was categorized in the “do do” group. Of the participants in the “can’t do group” 70.3% was categorized in the “do do” group.

The average physical performance measures for women were 406.6 (87.3) meter on the 6MWD, 8.4 (2.6) seconds for TUG, 11.6 (3.0) number of stands for CST, 21.4 (7.1) kg for HGS, and a median of 51.0 (31.0, 77.0) minutes in MVPA or VPA per day. The average physical performance measures for men were a median of 458.0 (383.0, 516.0) meter on the 6MWD, 7.3 (6.3, 8.3) seconds for TUG, 12.0 (11.0, 13.8) number of stands for CST, 38.0 (32.5, 44.0) kg for HGS, and a median of 78.0 (48.0, 111.0) minutes in MVPA or VPA per day. For men and women, the prevalence of sarcopenia was 0% and 0.3%, and of osteosarcopenia 0% and 0.3%, respectively.

### Correlation PA and PC

Our data showed a significant correlation between PA and PC for the total study population (*r* = 0.46; *p* < 0.01), as well for women and men separately (*r* = 0.475 (*p* < 0.01); *r* = 0.337 (*p* < 0.05), resp.). Further, our data showed significant correlation between PA and other measures of physical capacity for the total study population (*r* = − 0.39 for TUG (*p* < 0.01), *r* = 0.40 for CST (*p* < 0.01), and 0.28 for HGS (*p* < 0.01)).

### Distribution Over Quadrant Groups

**Fig. 1:**
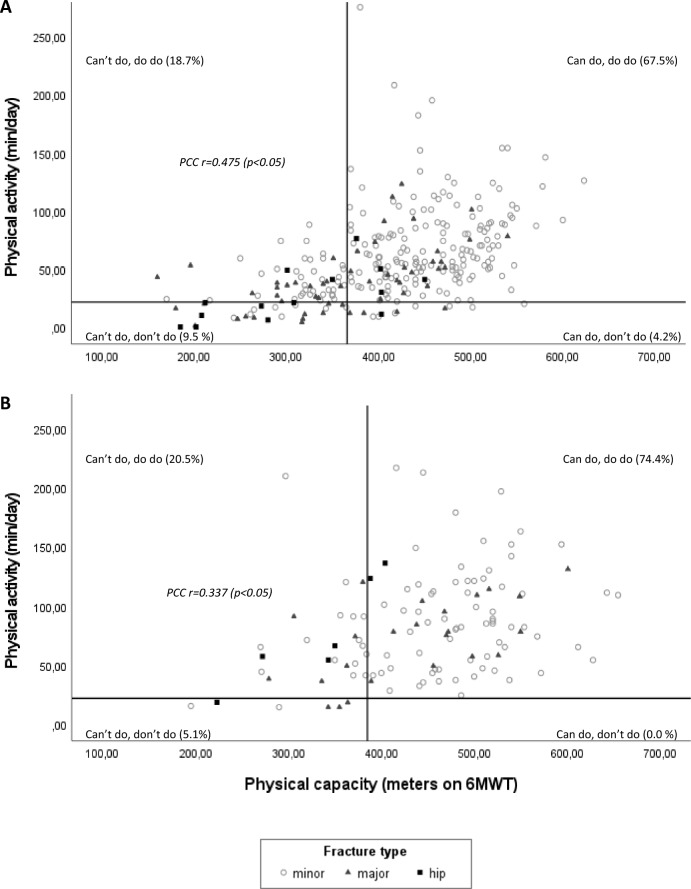
Overview of PC-PA quadrant framework for women (**A**) and men (**B**)

As shown in Fig. 1A and B, distribution of participants in quadrant groups for women and men, respectively, was as follows; 9.5% and 5.1% in the “can’t do, don’t do” group; 4.2% and 0% in the “can do, don’t do”; 18.7% and 20.5% in the “can’t do, do do” and 67.5% and 74.4% in the “can do, do do”. Supplementary Fig. 1A–C and 2A–C show the distribution over quadrant groups for minor, major and hip fractures, for women and men, respectively. For men and women together the distribution over the quadrants was as follows: 8.3% can’t do, don’t do”; 3.0% “can do, don’t do”; 19.3% “can’t do, do do”; 69.5% “can do, do do”. The odds of having poor PA was 9.76 (95% CI: 4.82–19.80) in participants with a poor PC as compared to preserved PC (total cohort).

6MWT, 6 Minute walking test; PCC, Pearson correlation coefficient. Fracture groups according to Center et al. [25]. Figure 1A displays the scatterplot of physical activity (measured by MOX accelerometer) and physical capacity (measured by 6-min walking test) of the female participants. The cut-off for low PC is 366 m (can’t do), the cut-off for low PA is 21 min/day (don’t do). The distribution over the four quadrant groups is as follows; 9.5% in the “can’t do, don’t do” group; 4.2% in the “can do, don’t do”; 18.7% in the “can’t do, do do” and 67.5% in the “can do, do do”. Pearson correlation coefficient is 0.475, *p* < 0.05. Figure 1B displays the scatterplot of physical activity and physical capacity for the male participants. The cut-off for low PC is 385 m (can’t do), the cut-off for low PA is 21 min/day (don’t do). Distribution over quadrants is as follows: 5.1% in the “can’t do, don’t do” group; 0% in the “can do, don’t do”; 20.5% in the “can’t do, do do” and 74.4% in the “can do, do do”. Pearson correlation coefficient is 0.337, *p* < 0.05.

### Characteristics of Quadrant Groups

As shown in Table 1, women in the “can’t do, don’t do” versus the “can do, do do” group were older, used alcohol less often, presented more often with hip or major fractures, were diagnosed more frequently with osteoporosis. Further, walking aids were used more frequently and women reported a higher of fear of falling and fall incidents in the past 12 months. Moreover, they performed worse on all physical performance tests. Comparable results were shown for the “can’t do, do do” group versus the “can do, do do” group, except for BMD measures and prevalent VF. The “can’t do, don’t do” compared to the “can’t do, do do” group presented with more hip fractures, and less minor fractures, more osteoporosis and a worse performance on TUG and PA measures. As shown in Table 2, men in the “can’t do, don’t do” group compared to the “can do, do do” group respectively, used less often alcohol, had less minor fractures, were diagnosed more frequently with osteoporosis, used walking aids more frequently, and performed worse on all functional performance tests. Similar results were seen for the “can’t do, do do” group versus the “can do, do do”, except for alcohol use and minor fractures.

**Table 1:** Patient characteristics of female quadrant groups

	Total population	Can’t do, don’t do	Can do, don’t do	Can’t do, do do	Can do, do do	Difference between groups
Number of patients (%)	283 (100)	27 (9.5)	12 (4.2)	53 (18.7)	191 (67.5)	*p* < 0.05*
Age, years^a^	64.5 (8.1)	70.4 (7.7)‡	67.4 (7.9)	67.7 (8.7)‡	62.6 (7.32)	*p* = 0.06
50–59^b^	90 (31.8)	2 (7.4)	2 (16.7)	13 (24.5)	73 (38.2)	
60–69	114 (40.3)	11 (40.7)	3 (25.0)	16 (30.2)	84 (44.0)	
70–79	67 (23.7)	11 (40.7	7 (58.3	19 (35.8)	30 (15.7)	
80 +	12 (4.2)	3 (11.1)	–	5 (9.4)	4 (2.1)	
BMI, kg/m^a^	27.4 (4.8)	27.4 (6.6)	26.2 (5.0)	28.9 (5.0)‡	27.0 (4.6)	
Current smokers^b^	178 (62.9)	16 (59.3)	4 (33.3)	36 (67.9)	122 (63.9)	*p* = 0.15
Alcohol use^b^	216 (76.3)	17 (63.0)‡	7 (58.3)	31 (58.5) ‡	161 (84.3)	*p* < 0.05*
Living alone^b^	61 (21.6)	6 (22.2)	1 (8.3)	16 (30.2)	38 (20.0)	*p* = 0.28
Fracture location^b^						*p* < 0.05*
Hip	14 (4.9)	7 (25.9)†‡	1 (8.3)	2 (3.8)	4 (2.1)	
Major	56 (19.8)	12 (44.4)‡	4 (33.3)	16 (30.2)‡	24 (12.6)	
Minor incl. finger and toe	213 (75.3)	8 (29.6)†‡	7 (58.3)	35 (66.0)‡	163 (85.3)	
Time since fracture (days)^a^	106.3 (29.3)	107.4 (37.4)	105.7 (30.3)	111.2 (30.6)	104.8 (27.7)	*p* = 0.57
Fracture caused by a fall^b^	346 (86.5)	23 (85.2)	11 (91.7)	46 (86.6)	168 (88.0)	*p* = 0.95
BMD^b^						*p* < 0.05*
Normal BMD	68 (24.0)	2 (7.4)	2 (16.7)	13 (24.5	51 (26.7)	
Osteopenia	145 (51.2)	10 (37.0)	5 (41.7)	31 (58.5)	99 (51.8)	
Osteoporosis	70 (24.7)	15 (55.6)†‡	5 (41.7)	9 (17.0)	41 (21.5)	
Hip T-score^a^	− 0.872	− 1.67 (0.94) †‡	− 1.14 (1.10)	− 0.82 (1.12)	− 0.77 (.97)	*p* < 0.05*
Vertebral T-score^a^	− 1.208	− 1.89 (1.50) †‡	− 1.97 (1.06)	− 0.94 (1.11)	− 1.14 (1.29)	*p* < 0.05*
Femoral neck T-Score^a^	− 1.451	− 2.25 (0.70)†‡	− 1.78 (1.11)	− 1.36 (0.96)	− 1.36 (0.98)	*p* < 0.05*
Prevalent Vertebral fracture Gr.2-3^b^	35 (12.4)	8 (29.6)‡	1 (8.3)	10 (18.9)	16 (8.4)	*p* < 0.05*
Comorbidities^b^						
Cardiovascular	113 (39.9)	16 (59.3)	2 (16.7)	28 (52.8)	67 (35.1)	*p* < 0.05*
Asthma/COPD	27 (9.5)	5 (18.5)	0 (0)	9 (17.0)	13 (6.8)	*p* < 0.05*
Osteoarthritis	33 (11.7)	5 (18.5)	2 (16.7)	7 (13.2)	19 (9.9)	*p* = 0.38
Diabetes mellitus	20 (9.5)	6 (22.2)‡	0 (0)	8 (15.1)	6 (3.1)	*p* < 0.05*
Mood/anxiety disorder	12 (4.2)	0 (0)	0 (0)	4 (7.5)	8 (4.2)	–
Glucocorticoid use^b^	12 (4.2)	2 (7.4)	0 (0)	3 (5.7)	7 (3.7)	*p* = 0.63
Urinary incontinence^b^	80 (28.5)	12 (44.4)	3 (25.0)	15 (28.3)	50 (26.5)	*p* = 0.28
Vision impairment^b^	242 (91.3)	25 (100)	11 (100)	46 (90.2)	160 (89.9)	–
Use of walking aids^b^	12 (4.2)	5 (18.5)‡	0 (0)	5 (9.4)‡	2 (1)	*p* < 0.05*
Two or more falls last year^b^	34 (12.1)	4 (14.8)	1 (8.3)	8 (15.1)	21 (11.1)	*p* = 0.78
Dizzyness/Balance problems^b^	73 (25.8)	10 (40.0)	5 (45.5)	19 (37.3)	39 (21.9)	*p* < 0.05*
Fear of falling^b^	37 (13.2)	8 (29.6)‡	0 (0)	14 (26.4) ‡	15 (8)	*p* < 0.05*
TUG, seconds^a^	8.4 (2.6)	13.0 (4.1)*†‡	7.6 (1.1)†	10.5 (2.1)‡	7.2 (1.0)	*p* < 0.05*
CST, no of stands^a^	11.6 (3.0)	8.6 (2.3)*‡	12 (2.1)†	9.5 (2.1)‡	12.6 (2.7)	*p* < 0.05*
HGS, kg^a^	21.4 (7.1)	17.9 (6.6)‡	19.5 (8.2)	19.4 (7.2)‡	22.5 (6.8)	*p* < 0.05*
6MWD, meters^a^	406.6 (87.3)	274.1 (55.8)*‡	436.4 (55.7)†	313.0 (45.9)‡	456.2 (54.2)	*p* < 0.05*
MVPA/VPA, av. min/day^a^	51.0 (31.0, 77.0)	11 (7, 18)†‡	12 (11.3, 17.8)†‡	36.0 (28.0, 48.5)‡	63.0 (47.0, 89.0)	*p* < 0.05*

Missing sample data per category: living alone *n* = 282; TH score *n* = 276; LS score *n* = 281; FN score *n* = 276; urinary incontinence *n* = 281; vision impairment *n* = 268; dizziness/balance problems *n* = 371; fear of falling *n* = 280; falling last year *n* = 282. In case of missing data valid percentages are reported

^a^continuous variable: mean (SD), median (IQR)

^b^categorical variable: number (%)

*BMI* body mass index, *BMD* bone mineral density, *TUG* timed up and go, *CST* 30 s chair stand test, *HGS* hand grip strength, *6MWD* six-minute walking distance, *MVPA* moderate-to-vigorous physical activity, *VPA* vigorous physical activity, *Av* average

*p < 0.05 vs can do, don’t do, †*p* < 0.05 vs can’t do, do do, ‡*p* < 0.05 vs can do, do do

**Table 2:** Patient characteristics of male quadrant groups

	Total population	Can’t do, don’t do	Can do, don’t do	Can’t do, do do	Can do, do do	Difference between groups
Number of patients *n* (%)^b^	117 (100)	6 (5.1)	0 (0)	24 (20.5%)	87 (74.4%)	*p* < 0.05*
Age, years^a^	65 (55.5, 72.0)	68.0 (62.0, 83.3)	–	69.0 (62.5, 77.8))‡	64.0 (55, 70)	*p* < 0.05*
50–59^b^	42 (35.9)	1 (16.7)		3 (12.5)	38 (43.7)	
60–69	39 (33.3)	3 (50)		9 (37.5)	27 (31.0)	
70–79	27 (23.1)	–		7 (29.2)	20 (31.0)	
80 +	9 (7.7)	2 (33.3)		5 (20.8)	2 (2.3)	
BMI, kg/m^a^	27.6 (25.3, 30.6)	27.6 (23.8, 28.5)	–	29.3 (26.0, 31.8)	27.4 (25.2, 30.2)	*p* = 0.23
Current Smokers^b^	91 (77,8)	5 (83.3)	–	18 (75)	68 (78.2)	*p* = 0.92
Alcohol use^b^	100 (85.5)	3 (50)‡	–	19 (79.2)	78 (89.7)	*p* < 0.05*
Living alone^b^	19 (16.2)	2 (40.0)	–	4 (16.7)	13 (15.1)	*p* = 0.29
Fracture location^b^						*p* < 0.05*
Hip	6 (5.1)	1 (16.7)		3 (12.5)	2 (2.3)	
Major	24 (20.5)	3 (50)		6 (25.0)	15 (17.2)	
Minor incl. finger and toe	87 (74.4)	2 (33.3) ‡		15 (62.5)	70 (80.5)	
Fracture caused by a fall^b^	98 (83.8)	5 (83.3)	–	20 (83.3)	73 (83.9)	*p* = 1.0
Time since fracture (days)^a^	107 (90.5, 131.5)	134.0 (94.0, 143.3)	–	111.0 (86.8, 123.8)	107.0 (90.0, 128.0)	*p* = 0.48
BMD^b^			–			*p* < 0.05*
Normal BMD	44 (37.6)	0 (0)		8 (33.3)	32.7 (41.4)	
Osteopenia	55 (47.0)	3 (50)		9 (37.5)	43 (49.4)	
Osteoporosis	18 (15.4)	3 (50.0) ‡		7 (29.2) ‡	8 (9.2)	
Hip T-score^a^	− 0.509	− 1.5 (− 1.9, − 0.6)		− 0.3 (− 1.4–0.0)	− 0.4, (− 1.0, 0.1)	*p* = 0.06
Vertebral T-score^a^	− 0.817	− 0.9 (− 2.7, − 0.5)		− 1.1 (− 1.1, − 0.8)	− 0.9 (− 1.6, 0.1)	*p* = 0.64
Femoral neck T-Score^a^	− 1.204	− 1.6 (− 2.7, − 1.4)		− 0.4 (− 2.5, − 0.4)	− 1.1 (− 1.7, − 0.6)	*p* = 0.07
Prevalent Vertebral fracture Gr.2-3^b^	14 (12.0)	2 (33.3)	–	5 (20.8)	7 (8.0)	*p* < 0.05*
Comorbidities^b^						
Cardiovascular	50 (42.7)	5 (83.3)		13 (54.2)	32 (36.8)	*p* < 0.05*
Asthma/COPD	13 (11.1)	2 (33.3)	–	3 (12.5)	8 (9.2)	*p* = 0.18
Osteoarthritis	12 (10.3)	1 (16.7)		3 (12.5)	8 (9.2)	*p* = 0.61
Diabetes mellitus	9 (7.7)	0 (0)			7 (8.0)	*p* = 1
Mood/anxiety disorder	5 (4.3)	2 (33.3)‡		1 (4.2)	2 (2.3)	*p* < 0.05*
Glucocorticoid use^b^	6 (5.1)	1 (16.7)	–	0 (0)	5 (5.7)	*p* = 0.26
Urinary incontinence^b^	14 (12.1)	1 (16.7)	–	6 (26.1)	7 (8.0)	*p* = 0.06
Vision impairment^b^	100 (94.3)	5 (100)	–	18 (81.8)‡	77 (97.0)	*p* < 0.05*
Walking aid^b^	3 (2.6)	1 (16.7)‡	–	2 (8.3)‡	0(0)	*p* < 0.05*
Two or more falls last year^b^	34 (14.5%)	0 (0)	–	6 (3.5%)	11 (12.6)	*p* = 0.16
Dizziness/balance problems^b^	21 (17.9)	2 (40.0)	–	6 (27.7)	13 (16.5)	*p* = 0.18
Fear of falling)^b^	2 (1.7)	0 (0)	–	1 (4.3)	1 (1.1)	*p* = 0.44
TUG, seconds^a^	7.3 (6.3, 8.3)	9.8 (8.8, 15.9)‡	–	9.5 (8.3, 10.9)‡	7.0 (6.0, 7.7)	*p* < 0.05*
30CST, no of stands^a^	12.0 (11.0, 13.8)	8.5 (7.5, 11.0)‡	–	10.5 (9.3, 11.8)‡	13 (11.8, 14.0)	*p* < 0.05*
HGS, kg^a^	38.0 (32.5, 44.0)	25.0 (24.0, 33.5)‡	–	38.0 (30.0, 40.0)‡	40.0 (34.0, 44.0)	*p* < 0.05*
6MWD, meters^a^	458.0 (383.0, 516.0)	316.5 (216.0, 357.3)‡	–	359.0 (309.0, 375.0)‡	486.0 (445.0, 527.0)	*p* < 0.05*
MVPA/VPA, av. min/day^a^	78.0 (48.0, 111.0)	14.5 (14.0, 18.0)†‡	–	65.5 (49.5, 91.0)‡	85 (57.0, 116.0)	*p* < 0.05*

Sample data per category in case of missing data: living alone *n* = 115; hip/vertebral/femoral neck T-score *n* = 116; dizziness/balance problems *n* = 106; urinary incontinence *n* = 116; fear of falling *n* = 116; vision impairment *n* = 108. In case of missing data valid percentages are reported

^a^continuous variable: mean (SD), median (IQR)

^b^categorical variable: number (%)

*BMI* body mass index, *BMD* bone mineral density, *TUG* timed up and go, *CST* 30 s chair stand test, *HGS* hand grip strength, *6MWD* six-minute walking distance, *MVPA* moderate-to-vigorous physical activity, *VPA* vigorous physical activity, *Av* average

†*p* < 0.05 vs can’t do, do do, ‡*p* < 0.05 vs can do, do do

## Discussion

This is the first study to implement the “can do, do do” framework in patients with a recent fracture attending the FLS for fracture risk evaluation. The framework offers additional insight on impaired physical function of FLS patients by categorizing them into subgroups of physical activity and capacity measures. It enables identification of FLS patients with definable treatment traits and, thus, might be useful in stratification for targeted interventions in the future.

### “Can Do, Do Do” Framework in FLS Patients

Correlation between PC measures (6MWT, TUG, CST and HGS) and PA was significant, but low. This is in line with findings of Van Lummel et al. and Koolen et al., who concluded that these measures are related but separate domains of physical functioning [10, 22]. The low proportion of participants with poor PA (less than 21,4 min/day) in our study population is surprising, especially since approximately 50% of the Dutch general population of 50 years and older do not meet the WHO recommendations of a minimal of 150 min (M)VAP per week [41]. Remarkably, the inhabitants of the region of the Netherlands in which the hospital is located (North-Limburg) have a poorer health status defined as the total number of chronic diseases including COPD, smoking, BMI compared to the general Dutch population [42]. A possible explanation for the high proportion of preserved PA is that participants were aware of the activity recording during accelerometer measurement and consequently could increase activity during the test period. Also, physical activity of the Dutch population was not measured by accelerometer, but with the SQUASH questionnaire making results less comparable. Furthermore, this population might be subject to a healthy cohort bias; first, only 60% of all consecutive patients with a clinical fracture attended the FLS for fracture risk evaluation. FLS attenders are less frail, and have less hip fractures compared to patients that do not respond to the FLS invitation [43, 44]. Second, participation in the FX MoVie study was voluntary, and compared to the FLS attenders who did not participate, FX MoVie participants were younger, had less severe fracture types and less prevalent VFs [3]. This healthy complier bias is also reflected in the low percentage of sarcopenia in our population. The prevalence of (osteo)sarcopenia in this study is very low. This was mostly due to the prevalence of low ALM/m2 in our total population of only 0.8%, as opposed to the proportion of patients that scored below their cut-off points on HGS and/or CST of 17.6% and 18.5%, respectively. Furthermore, compared to many other guidelines, the EWGSOP2 identifies sarcopenia less often, pointing to a possible underestimation of sarcopenia [45, 46].

The application of the “can do, do do” concept strongly relies on the pre-determined cut-off scores. The cut-off for low physical activity (21.4 min (MVPA/day) was derived from the lower end of amount of 150–300 min(M)VPA/week recommended by the WHO guidelines [13] and was, among others, based on a review that showed a maximal risk reduction for mortality at MVPA > 24 min/day [47]. A study of Buchner et al. revealed a higher falls rate for women with a MVPA of less than 25.1 min/day (lowest compared with highest quartile of MVPA) [48]. However, changing our cut-off value from 21.4 to 25 min/day, merely increased the proportion of patients with low PA from 11 to 15%. Furthermore, we did not take into account if MVPA was performed in bouts of at least 10 min uninterrupted PA, which might lead to an overestimation of MVPA of the participants. However, physical activity of any bout duration is associated with improved health outcomes and the WHO no longer recommends it [13]. Lastly, the MOX accelerometer cut-off points to categorize dynamic activity were validated in a younger population [29]. However, this would have resulted in an underestimation instead of an overestimation of MVPA.

The six-minute walking showed that participants who “can’t do” had an almost tenfold higher odds to be categorized in the “don’t do” group, compared to the “can do” group. Only 3% of all the “don’t do” participants were categorized in the “can do, don’t do” group, meaning they were able to be physical active, but did not undertake physical activity. This group encompassed only 4% of all “can do” participants. These results indicate that if the FLS population “can do”, they most often “do do”.

About 20% of FLS participants “can’t do” but “do do” anyway, indicating that this group might exhibits risky behavior that could result in a higher rate of falls. This “can’t do, do do” group had significantly lower measures on all functional capacity tests, compared to those in the “can do, do do” group. They were relatively older, had higher fear of falling and a higher proportion used a walking aids. These are all known fall risk factors [15]. Older adults with poor gait and balance might be less equipped to prevent falls against more severe perturbations of balance during PA [15], and have been proven to have higher rate of falls during habitual walking [19]. Combining this with a high exposure to mishaps during PA, this “can’t do, do do” group might be prone to future falls [15, 19]. However, this group might also benefit from their preserved daily activity in several ways; first, physical activity can improve bone strength and offers benefit in the management of osteoporosis [49]. In line with the literature, the “can’t do, do do” group had lower levels of osteoporosis and hip fractures compared to the “can’t do, don’t do” group. Second, low physical activity can lead to a decline in (instrumental) activities of daily living, balance and strength causing a vicious circle of declined physical functioning [50, 51]. This is in line with a study of Delbaere et al. used an approach similar to the “Can do, do do” framework to assess physiological fall risk (estimated using the physiological profile assessment) and perceived fall risk (measured by the falls efficacy scale international) in older community dwelling adults [52]. Both measures were associated with future falls. However, the group with high physiological but low perceived fall risk had a fall incidence of 30% during follow-up, which was lower compared to those with also a high perceived fall risk (41%). A possible explanation for this lower risk was that a positive outlook on life, community participation and preserved PA might be protective of future falls. Interestingly, Delbaere et al. did not find excessive risk-taking behavior based on the psychological profile in this group. However, the “can’t do, do do” group might be prone to reduce PA in the future, as falls and increased fear of falling have shown to be important factors to restrict PA, resulting in a vicious cycle of functional decline [15]. Attention to physical functioning to preserve PA and increase PC in this group at the FLS is therefore essential.

Only 8% of all participants were categorized in the “can’t do, don’t do” group. As expected, the largest differences in fall risk factors were seen between this group and the “can do, do do group”; the first had lower functional capacity measures and higher use of walking aid (men and women) and fear of falling (women). For women the group was older and had higher proportion of hip, major and prevalent vertebral fracture, both men and women had higher proportion of osteoporosis. These findings are consistent with literature that describes a decrease of functional performance with higher age and after major and hip fractures [53] and suggest that this group might be suitable for exercise interventions [16]. These interventions have shown positive effect on fall rate [16] and on prevention of fall-related injuries, specifically in patients at high risk for falling and patients with osteoporosis [54]. Moreover, the higher proportion of osteoporosis and VF (women) suggests that this subgroup could benefit from additional physical activity training in a controlled setting to increase bone strength, in addition to anti-osteoporotic treatment [49]. Current WHO recommendations state that the benefits of PA outweighed the potential harms, and stated that possible harms can be managed by a gradual increase in the amount and intensity of physical activity [13].

### Strengths and Limitations

Our study has several strengths. This is a large, real-life sample of patients with a recent fracture attending the FLS, including a wide range of fracture types, with and without osteoporosis. Participants were comprehensively assessed allowing for comparison of multiple fall risk variables and extensive physical functioning measures between quadrant groups. However, several limitations must be addressed. As stated before, selection bias is present resulting in a relatively healthy study population. Further, due to the cross-sectional design of our study causality between clinical characteristics, quadrant group and fall or fracture risk cannot be evaluated. It is therefore essential that future research on this framework should focus on prospective falls and fall-related injury in these quartiles. Furthermore, these future studies should include more falls specific PC measures such as reactive balance, as this might contribute to the discrimination between fallers and non-fallers [55]. Moreover, PA and PC measurements could be affected by the type of fracture the participant sustained four months before the FLS visit and measurements. However it is also plausible that PC and PA contributed to the fall and fracture incident itself. Lastly, as stated before, cut-off scores are of large influence and application of the concept should always be considered within their limitations.

## Conclusion

The “can do, do do” framework offers a new approach to understanding PC a PA measures in fracture patients. Moreover, it can be used to categorize patients into subgroups with distinct clinical characteristics among which fall and fracture risk factors. Results of this FLS population showed that patients that “can do”, “do do”, reducing the population eligible for behavioral interventions. Nevertheless, 20% of the FLS patients “can’t do” but “do do” while they have a high prevalence of fall risk factors compared to persons that “can do” and “ do do”, which may indicate a group prone for falling. Prospective studies are needed to assess the relationship between quartile categorization and prospective fall and fracture events.

## Supplementary Information

Below is the link to the electronic supplementary material.

Supplementary file1 (DOCX 168 KB)

## Data Availability

Research data are not shared due to privacy or ethical restrictions.

## References

[1] Rubenstein LZ, Josephson KR (2002). The epidemiology of falls and syncope. Clin Geriatr Med.

[2] Schwenk M, Lauenroth A, Stock C, Moreno RR, Oster P, McHugh G, Todd C, Hauer K (2012). Definitions and methods of measuring and reporting on injurious falls in randomised controlled fall prevention trials: a systematic review. BMC Med Res Methodol.

[3] Vranken L, Wyers CE, Van der Velde RY (2022). Association between incident falls and subsequent fractures in patients attending the fracture liaison service after an index fracture: a 3-year prospective observational cohort study. BMJ Open.

[4] van Geel TA, van Helden S, Geusens PP, Winkens B, Dinant GJ (2009). Clinical subsequent fractures cluster in time after first fractures. Ann Rheum Dis.

[5] Lems WF, Dreinhöfer KE, Bischoff-Ferrari H (2017). EULAR/EFORT recommendations for management of patients older than 50 years with a fragility fracture and prevention of subsequent fractures. Ann Rheum Dis.

[6] van den Bergh JP, van Geel TA, Geusens PP (2012). Osteoporosis, frailty and fracture: implications for case finding and therapy. Nat Rev Rheumatol.

[7] Li N, Hiligsmann M, Boonen A, van Oostwaard MM, de Bot R, Wyers CE, Bours SPG, van den Bergh JP (2021). The impact of fracture liaison services on subsequent fractures and mortality: a systematic literature review and meta-analysis. Osteoporos Int.

[8] Vranken L, Wyers CE, Van der Velde RY, Janzing HM, Kaarsemaker S, Geusens PP, Van den Bergh JP (2018). Comorbidities and medication use in patients with a recent clinical fracture at the Fracture Liaison Service. Osteoporos Int.

[9] Van Helden S, van Geel AC, Geusens PP, Kessels A, Kruseman ACN, Brink PR (2008). Bone and fall-related fracture risks in women and men with a recent clinical fracture. JBJS.

[10] van Lummel RC, Walgaard S, Pijnappels M, Elders PJ, Garcia-Aymerich J, van Dieën JH, Beek PJ (2015). Physical performance and physical activity in older adults: associated but separate domains of physical function in old age. PLoS ONE.

[11] Rikli RE, Jones CJ (1998). The reliability and validity of a 6-minute walk test as a measure of physical endurance in older adults. J Aging Phys Act.

[12] Caspersen CJ, Powell KE, Christenson GM (1985). Physical activity, exercise, and physical fitness: definitions and distinctions for health-related research. Public Health Rep.

[13] World Health Organization (2020). WHO guidelines on physical activity and sedentary behaviour.

[14] Cöster ME, Karlsson M, Ohlsson C, Mellström D, Lorentzon M, Ribom E, Rosengren B (2020). Physical function tests predict incident falls: a prospective study of 2969 men in the swedish osteoporotic fractures in men study. Scand J Public Health.

[15] Ambrose AF, Paul G, Hausdorff JM (2013). Risk factors for falls among older adults: a review of the literature. Maturitas.

[16] Sherrington C, Fairhall N, Kwok W, Wallbank G, Tiedemann A, Michaleff ZA, Ng C, Bauman A (2020). Evidence on physical activity and falls prevention for people aged 65+ years: systematic review to inform the WHO guidelines on physical activity and sedentary behaviour. Int J Behav Nutr Phys Act.

[17] Heesch KC, Byles JE, Brown WJ (2008). Prospective association between physical activity and falls in community-dwelling older women. J Epidemiol Community Health.

[18] Klenk J, Kerse N, Rapp K, Nikolaus T, Becker C, Rothenbacher D, Peter R, Denkinger MD (2015). Physical activity and different concepts of fall risk estimation in older people-results of the ActiFE-Ulm study. PLoS ONE.

[19] Okubo Y, Seino S, Yabushita N, Osuka Y, Jung S, Nemoto M, Figueroa R, Tanaka K (2015). Longitudinal association between habitual walking and fall occurrences among community-dwelling older adults: analyzing the different risks of falling. Arch Gerontol Geriatr.

[20] Boyé ND, Mattace-Raso FU, Van der Velde N (2014). Circumstances leading to injurious falls in older men and women in the Netherlands. Injury.

[21] Lu Z, Lam FMH, Leung JCS, Kwok TCY (2020). The u-shaped relationship between levels of bouted activity and fall incidence in community-dwelling older adults: a prospective cohort study. J Gerontol A Biol Sci Med Sci.

[22] Koolen EH, van Hees HW, van Lummel RC, Dekhuijzen R, Djamin RS, Spruit MA, Van’t Hul AJ (2019). “Can do” versus “do do”: a novel concept to better understand physical functioning in patients with chronic obstructive pulmonary disease. J Clin Med.

[23] Dutch Institute for Healthcare Improvement CBO (2011). Richtlijn Osteoporose en Fractuurpreventie, Derde Herziening [Dutch] Utrecht.

[24] World Health Organisation (2004). ICD-10: international statistical classification of diseases and related health problems: tenth revision.

[25] Center JR, Nguyen TV, Schneider D, Sambrook PN, Eisman JA (1999). Mortality after all major types of osteoporotic fracture in men and women: an observational study. Lancet.

[26] Overgaard JA, Larsen CM, Holtze S, Ockholm K, Kristensen MT (2017). Interrater reliability of the 6-minute walk test in women with hip fracture. J Geriatr Phys Ther.

[27] Singh SJ, Puhan MA, Andrianopoulos V (2014). An official systematic review of the European Respiratory Society/American Thoracic Society: measurement properties of field walking tests in chronic respiratory disease. Eur Respir J.

[28] Bijnens W, Aarts J, Stevens A, Ummels D, Meijer K (2019). Optimization and validation of an adjustable activity classification algorithm for assessment of physical behavior in elderly. Sensors (Basel).

[29] van der Weegen S, Essers H, Spreeuwenberg M, Verwey R, Tange H, de Witte L, Meijer K (2015). Concurrent validity of the MOX activity monitor compared to the ActiGraph GT3X. Telemed J E Health.

[30] Beekman E, Mesters I, Gosselink R, Klaassen MP, Hendriks EJ, Van Schayck OC, de Bie RA (2014). The first reference equations for the 6-minute walk distance over a 10 m course. Thorax.

[31] Podsiadlo D, Richardson S (1991). The timed “Up & Go”: a test of basic functional mobility for frail elderly persons. J Am Geriatr Soc.

[32] Jones CJ, Rikli RE, Beam WC (1999). A 30-s chair-stand test as a measure of lower body strength in community-residing older adults. Res Q Exerc Sport.

[33] Ozcan Kahraman B, Ozsoy I, Akdeniz B, Ozpelit E, Sevinc C, Acar S, Savci S (2020). Test-retest reliability and validity of the timed up and go test and 30-second sit to stand test in patients with pulmonary hypertension. Int J Cardiol.

[34] Gill SD, de Morton NA, Mc Burney H (2012). An investigation of the validity of six measures of physical function in people awaiting joint replacement surgery of the hip or knee. Clin Rehabil.

[35] Roberts HC, Denison HJ, Martin HJ, Patel HP, Syddall H, Cooper C, Sayer AA (2011). A review of the measurement of grip strength in clinical and epidemiological studies: towards a standardised approach. Age Ageing.

[36] Cruz-Jentoft AJ, Bahat G, Bauer J (2019). Sarcopenia: revised European consensus on definition and diagnosis. Age Ageing.

[37] World Health Organisation (2003). Prevention and management of osteoporosis.

[38] Hirschfeld HP, Kinsella R, Duque G (2017). Osteosarcopenia: where bone, muscle, and fat collide. Osteoporos Int.

[39] Genant HK, Wu CY, van Kuijk C, Nevitt MC (1993). Vertebral fracture assessment using a semiquantitative technique. J Bone Miner Res.

[40] Hinkle DE, Wiersma W, Jurs SG (2003). Applied statistics for the behavioral sciences.

[41] Centraal Bureau voor de Statistiek (CBS) Leefstijl en (preventief) gezondheidsonderzoek; persoonskenmerken (2021). Accessed 15 June 2022

[42] GGD’en CBS en RIVM (2020) Gezondheidsmonitor Volwassenen en Ouderen (2020). Accessed 6 July 2022

[43] van den Berg P, van Haard PMM, Geusens PP, van den Bergh JP, Schweitzer DH (2019). Challenges and opportunities to improve fracture liaison service attendance: fracture registration and patient characteristics and motivations. Osteoporos Int.

[44] Eekman DA, van Helden SH, Huisman AM, Verhaar HJ, Bultink IE, Geusens PP, Lips P, Lems WF (2014). Optimizing fracture prevention: the fracture liaison service, an observational study. Osteoporos Int.

[45] Stuck AK, Mäder NC, Bertschi D, Limacher A, Kressig RW (2021). Performance of the EWGSOP2 cut-points of low grip strength for identifying sarcopenia and frailty phenotype: a cross-sectional study in older inpatients. Int J Environ Res Public Health.

[46] Harvey NC, Orwoll E, Kwok T (2021). Sarcopenia definitions as predictors of fracture risk independent of FRAX(®), falls, and BMD in the osteoporotic fractures in men (MrOS) study: a meta-analysis. J Bone Miner Res.

[47] Ekelund U, Tarp J, Steene-Johannessen J (2019). Dose-response associations between accelerometry measured physical activity and sedentary time and all cause mortality: systematic review and harmonised meta-analysis. BMJ.

[48] Buchner DM, Rillamas-Sun E, Di C (2017). Accelerometer-measured moderate to vigorous physical activity and incidence rates of falls in older women. J Am Geriatr Soc.

[49] Brooke-Wavell K, Skelton DA, Barker KL (2022). Strong, steady and straight: UK consensus statement on physical activity and exercise for osteoporosis. Br J Sports Med.

[50] Deshpande N, Metter EJ, Lauretani F, Bandinelli S, Guralnik J, Ferrucci L (2008). Activity restriction induced by fear of falling and objective and subjective measures of physical function: a prospective cohort study. J Am Geriatr Soc.

[51] Ambrose AF, Cruz L, Paul G (2015). Falls and fractures: a systematic approach to screening and prevention. Maturitas.

[52] Delbaere K, Close JC, Brodaty H, Sachdev P, Lord SR (2010). Determinants of disparities between perceived and physiological risk of falling among elderly people: cohort study. BMJ.

[53] Szulc P (2020). Impact of bone fracture on muscle strength and physical performance-narrative review. Curr Osteoporos Rep.

[54] Zhao R, Bu W, Chen X (2019). The efficacy and safety of exercise for prevention of fall-related injuries in older people with different health conditions, and differing intervention protocols: a meta-analysis of randomized controlled trials. BMC Geriatr.

[55] McCrum C (2020). Fall prevention in community-dwelling older adults. N Engl J Med.

