# Supplementary material for: Physical Functioning in Patients with a Recent Fracture: The “Can Do, Do Do” Framework Applied to Explore Physical Capacity, Physical Activity and Fall Risk Factors
Source: Calcif Tissue Int. 2023 Jun 27;113(2):195–206. doi: 10.1007/s00223-023-01090-3 (PMC10371931; doi:10.1007/s00223-023-01090-3)
Supplement: Supplementary file 1 — Supplementary file1 (DOCX 168 KB) [file 223_2023_1090_MOESM1_ESM.docx]

**Supplemental figure 1A-C - Overview of PC-PA quadrant framework for women stratified by fracture type**


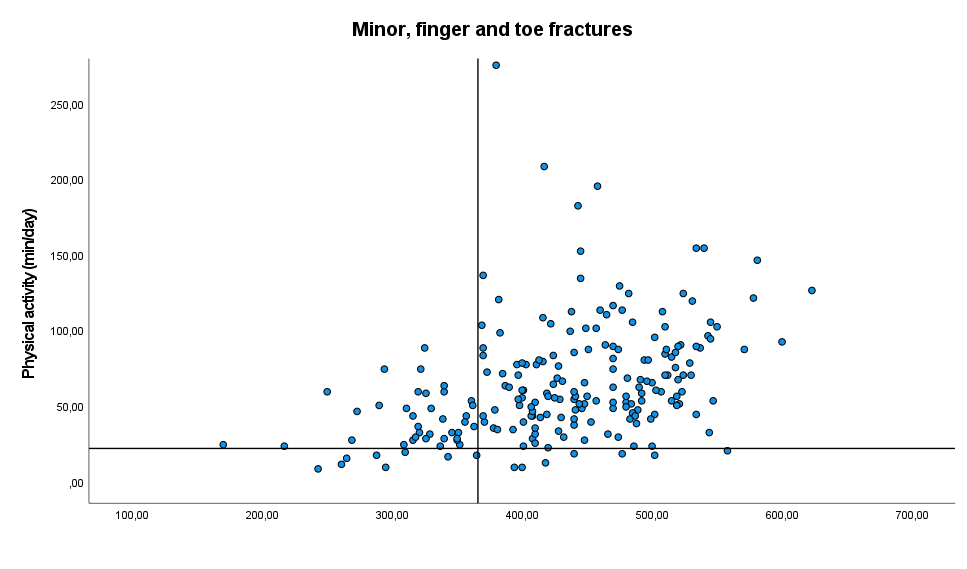


**A**

**B**

**C**

Can do, do do (76.5%)

Can’t do, do do (16.4%)

Can do, don’t do (3.3%)

Can’t do, don’t do (3.8%)


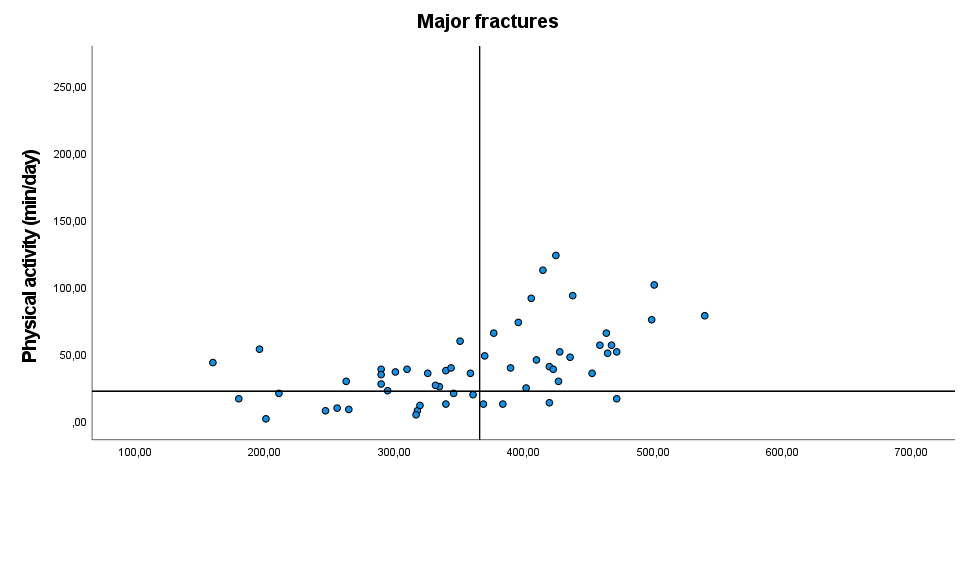


Can do, do do (42.9%)

Can’t do, do do (28.6%)

Can do, don’t do (7.1%)

Can’t do, don’t do ( 21.4%)


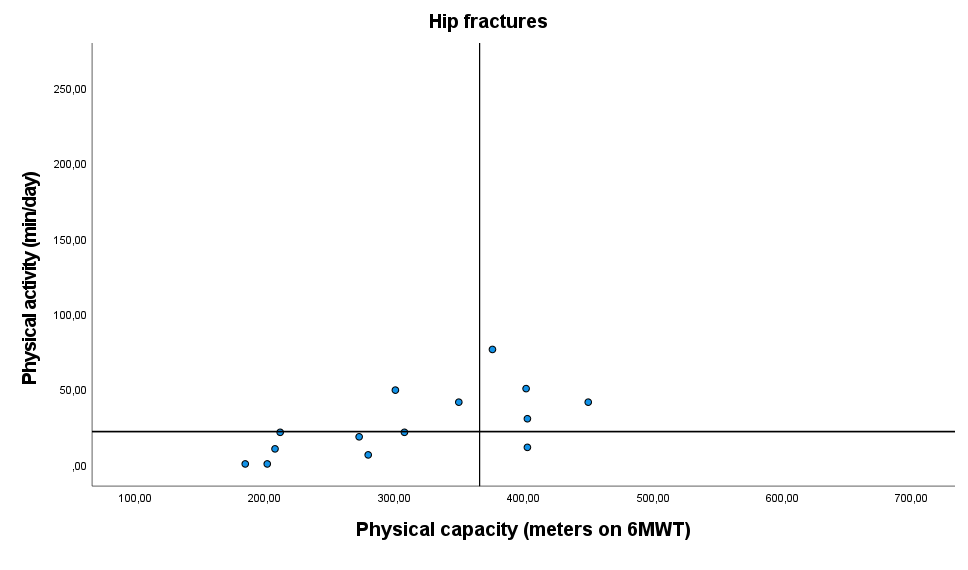


Can do, do do (28.6%)

Can’t do, do do (14.3%)

Can do, don’t do (7.1%)

Can’t do, don’t do (50%)

**Supplemental figure 2A-C - Overview of PC-PA quadrant framework for men stratified by fracture type**


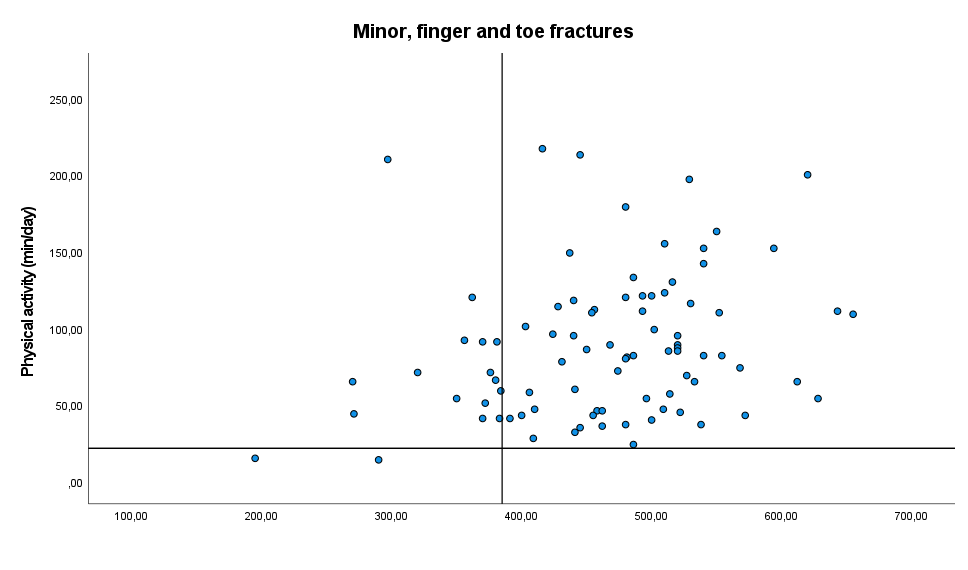


**A**

**B**

**C**


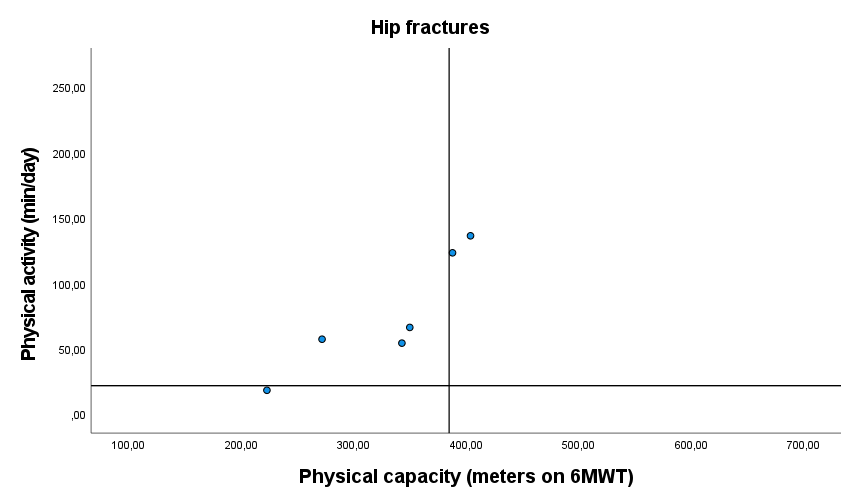

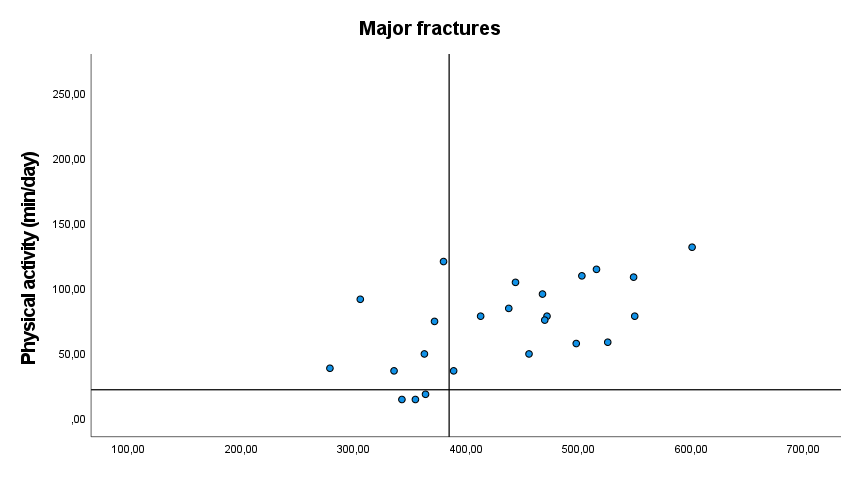


Can do, do do (33.3%)

Can do, do do (62.5%)

Can do, do do (80.5%)

Can’t do, do do (50.0%)

Can’t do, don’t do (16.7%)

Can’t do, do do (25%)

Can do, don’t do (0%)

Can do, don’t do (0%)

Can’t do, don’t do (12.5%)

Can’t do, don’t do (2.3%)

Can do, don’t do (0%)

Can’t do, do do (17.2%)
